# Nucleotide-Binding Domain Leucine-Rich Repeat Containing Proteins and Intestinal Microbiota: Pivotal Players in Colitis and Colitis-Associated Cancer Development

**DOI:** 10.3389/fimmu.2018.01039

**Published:** 2018-05-14

**Authors:** Anna Prossomariti, Harry Sokol, Luigi Ricciardiello

**Affiliations:** ^1^Department of Medical and Surgical Sciences, University of Bologna, Bologna, Italy; ^2^Center for Applied Biomedical Research (CRBA), S. Orsola-Malpighi Hospital, University of Bologna, Bologna, Italy; ^3^Sorbonne Université, École normale supérieure, PSL Research University, CNRS, INSERM, AP-HP, Hôpital Saint-Antoine, Laboratoire de biomolécules, LBM, Paris, France; ^4^INRA, UMR1319 Micalis & AgroParisTech, Jouy en Josas, France

**Keywords:** nucleotide-binding domain leucine-rich repeat containing signaling, microbiota, intestinal inflammation, ulcerative colitis, colitis-associated cancer

## Abstract

The nucleotide-binding domain leucine-rich repeat containing (NLR) proteins play a fundamental role in innate immunity and intestinal tissue repair. A dysbiotic intestinal microbiota, developed as a consequence of alterations in NLR proteins, has recently emerged as a crucial hit for the development of ulcerative colitis (UC) and colitis-associated cancer (CAC). The concept of the existence of functional axes interconnecting bacteria with NLR proteins in a causal role in intestinal inflammation and CAC aroused a great interest for the potential development of preventive and therapeutic strategies against UC and CAC. However, the most recent scientific evidence, which highlights many confounding factors in studies based on microbiota characterization, underlines the need for an in-depth reconsideration of the data obtained until now. The purpose of this review is to discuss the recent findings concerning the cross talk between the NLR signaling and the intestinal microbiota in UC and CAC development, and to highlight the open issues that should be explored and addressed in future studies.

## Introduction

Under physiological conditions, a delicate balance between immune activation and self-tolerance, resulting from a beneficial mutualistic relationship with the host gut microbial community, is responsible for maintaining a healthy intestinal homeostasis. Indeed, the ability of the host immune system to discriminate between potential harmful stimuli (i.e., pathogenic microbes, exogenous dangerous substances, and general cellular stress signals), and harmless signals (i.e., commensal gut microbiota and normal host-derived molecules), represents a sophisticated mechanism for preserving the intestinal epithelial integrity (1). The impairment of this host defense, allowing the overgrowth of pathogenic bacteria and weakening the intestinal barrier function, favors the onset of a pro-inflammatory and pro-tumorigenic microenvironment (2). To avoid the development of unfavorable pathological conditions, the host innate immune system is naturally able to recognize specific microbial conserved structures, named pathogen-associated molecular patterns (PAMPs), and to sense microbial molecules that characterize macrophages, neutrophils, monocyte, dendritic cells, and epithelial cells through a subset of pattern recognition receptors (PRRs) (3). PRRs families can be classified as transmembrane receptors, including toll-like receptors and C-type lectins receptors, responsible for recognizing extracellular and endosomal-derived PAMPs; and cytosolic receptors including retinoic acid-inducible gene-I-like receptors and nucleotide-binding domain leucine-rich repeat containing (NLRs) proteins, involved in the intracellular surveillance toward infections and recognition of self-derived damage-associated molecular patterns (DAMPs) (4). Alterations of the NLR family members have recently emerged as critically involved in the gut microbiota regulation, as well as in the genesis of colonic inflammation and colitis-associated tumorigenesis (5, 6). Here, we describe the recent knowledge on the relationship between NLR alterations and microbiota in the pathogenesis of colitis and colitis-associated cancer (CAC).

## NLR Family: An Overview

The NLR family comprises more than 20 intracellular immune receptors sharing structural domains with functional specialization. The central nucleotide-binding domain (NACHT), common to all NLR family members, is responsible for the activation of signaling complexes mediating the essential self ATP-dependent oligomerization (7). Differently, the C-terminal leucine-rich repeats domains and the N-terminal pyrin domains (PYD), or Caspase recruitment domain (CARD) are implicated in the recognition of PAMPs, and in autoregulation or in homotypic protein–protein interactions, respectively. According to their N-terminal domains, NLRs can be classified in different subfamilies: (a) the NLRA subfamily consisting of CIITA, which have an N-terminal CARD domain followed by an acid transactivation domain (AD), (b) the NLRB subfamily represented by NAIP which has a baculoviral inhibitory repeat-like domain, (c) the NLRP subfamily including NLRP1–14 which have an N-terminal PYD, and (d) the NLRC/X subfamily which includes proteins having an N-terminal CARD domain, also known as NODs (NOD1/NLRC1, NOD2/NLRC2) and NLRC4 (or IPAF) or an undefined domain (NOD3/NLRC3, NOD4/NLRC5, NOD5/NLRX1) (6). NLR receptors expression in the gastrointestinal tract was often recognized in both hematopoietic and non-hematopoietic cell lineages. All the members of NLR family cooperate in the innate immunity through different mechanisms including NF-kB and MAPK induction (8), or by triggering the Caspase-1 (Casp1) activation through the generation of multimeric signaling platforms named “inflammasomes” whose final effect is the processing and the maturation of IL-1β, IL-18, and IL-33 (9, 10). NOD1 and NOD2 contribute to the cytosolic surveillance in the presence of specific peptidoglycan fragments originating during cytosolic bacterial cell divisions in infected immune cells or as a result of bacterial wall degradation in host lysozymes, and act mainly through NF-kB or MAPK signaling activation (11). To date, the knowledge of NOD3/NLRC3 and NOD4/NLRC4 functions is very poor. On the other hand, NOD5/NLRX1 represents the only member of NLR family with a mitochondrial localization and a putative role in mitochondrial antiviral immunity (12) and in the modulation of the mitochondrial reactive oxygen species production (13). As mentioned above, NLR signaling does not evolve exclusively through the activation of NF-kB and MAPK pathways but also through the formation, under appropriate stimuli, of inflammasomes. Several NLR family members seem to be able to have potential inflammasome activity, however, the best-characterized NLRs inflammasomes are NLRP1, NLRP3, NLRC4 (or IPAF), NLRP6, and NLRP12. A multiplicity of different PAMPs or host-derived DAMPs are able to stimulate and activate NLRP3 inflammasome (14). Under these conditions, the NLRP3 oligomerization recruits the ASC (PYCARD) adaptor protein through the homotypic interaction between their PYDs. Similarly, the ASC protein, acting as a molecular bridge, makes its CARD domain available for the interaction with the same domain present in pro-Casp1. This event allows the autocleavage of pro-Casp1 and the subsequent formation of mature and active Casp1 which promotes the proteolitic processing and subsequently maturation of pro-inflammatory cytokines IL-1β and IL-18 in their biologically active state and their externalization (15). An important role for NLRP3 inflammasome and its dysregulation has been defined not only in microbial host-response but also in metabolic dysfunction (16, 17), inflammatory bowel disease (IBD), and colorectal cancer (18). Differently from NLRP3, NLRP1-mediated Casp1 activation does not require the recruitment of ASC: this can occur directly through the physical interaction between a CARD domain present in a C-terminal extension of NLRP1 and a classical CARD domain in pro-Casp1, although it is known that the involvement of ASC in the complex is able to enhance this inflammasome activity (19). The precise mechanisms underlying NLRP1 activation are still partially known. Differently from other inflammasomes, the NLRC4 can induce Casp1 activity with ASC-dependent or -independent (8) mechanisms. NLRC4 is generally activated by Gram-negative bacteria and specifically induced by cytosolic flagellin (20) and components of type III or IV secretion systems (21). More recently, NLRP6, another member of NLR family, has been found to be able to generate inflammasomes (22). It has been reported that NLRP6 can induce the maturation of IL-1β and the activation of NF-kB signaling assembling ASC and pro-Casp1 proteins in a classical molecular platform in response to DAMPs (23). Finally, the function of NLRP12, also known as Monarch1 protein, is recently emerging as a crucial negative regulator of canonical and non-canonical NF-kB signaling (24, 25) and a positive regulator of dendritic and myeloid cell migration (26).

## Alterations of NLR Proteins as Drivers of Intestinal Inflammation and CAC: Evidence from Pre-Clinical Models

Although it is widely accepted that a defective intestinal barrier function coupled with an inappropriate host–microbial interactions could promote colonic inflammation and CAC (2), the contribution of the NLR signaling to the intestinal homeostasis, colitis, and CAC is emerging in the latest years. In particular, a protective role of different inflammasome components on colitis and CAC has been observed. One of the first evidence supporting this hypothesis was provided by Dupaul-Chicoine and colleagues. They showed that *Casp1*^−/−^ and *Asc*^−/−^ mice were more susceptible to dextran sulfate sodium (DSS)-induced colitis (an acute model of epithelial injury) than *wild-type* (*wt*) mice and that exogenous administration of Il-18 rescued this phenotype, proving a fundamental role of Il-18 for tissue repair after injury (27). At the same time Allen et al. showed a critical disease outcome and increased tumor burden in the same *Casp1*^−/−^ and *Asc*^−/−^ mice in the DSS and azoxymethane (AOM)-DSS models of colitis and CAC (28). Interestingly, the enhanced tumor incidence encountered in *Asc*^−/−^ mice was associated with a significant attenuation of Il-1β and Il-18 levels in the colon within the tumor tissue. Moreover, compared to *wt* mice, a significant increase in inflammatory features, tumor multiplicity, and max tumor size was found in AOM-DSS-treated *Nlrp3*^−/−^ mice, but not in *Nlrc4*^−/−^ mice. These results led the authors to hypothesize a negative regulator function of Nlrp3 inflammasome during colitis and CAC (28). The role of *Asc, Nlrp3*, and *Casp1* deficiency, in the increased susceptibility to CAC was also simultaneously investigated by Zaki et al. In all three genotypes (*Asc*^−/−^, *Nlrp3*^−/−^, and *Casp1*^−/−^), the tumor burden was significantly higher compared to *wt* mice. The increased tumor incidence found in *Nlrp3*^−/−^ and *Casp1*^−/−^ mice correlated with an important local reduction of Il-18 levels and an increased colonic infiltration of macrophages. Importantly, the authors also found that the protective effect of Il-18 against CAC were associated with its ability to induce the tumor suppressors Ifn-γ and Stat3 (29). In agreement with the previously reported data, Hu et al. observed an enhanced tumor formation in *Casp1* deficient mice subjected to AOM-DSS through a modulation of proliferation and apoptosis in colonic epithelial cells (30). However, differently from Allen et al., they also demonstrated that *Nlrc4*^−/−^ mice showed significantly increased tumor numbers and load compared to *wt* mice, with tumors characterized by increased aggressiveness and invasive potential. Importantly, the authors did not observe differences in colonic inflammation severity induced by DSS (30). These results suggest a role of Nlrc4 in the protection against CAC. By contrast, a recent study by Zhou and colleagues showed that Nlrp3 inhibition by the flavonoid oroxylin A may have beneficial effects against colitis in DSS-treated mice (31).

More recent evidence also supports a protective role of *Nlrc3* on CAC onset. Karki and colleagues found a significant increased number of colonic lesions in *Nlrc3*^−/−^ mice treated with AOM-DSS compared to *wt* mice. The increased tumor number was associated with a colonic upregulation of inflammatory mediators (including Il-1β, Il-6, Tnf, G-csf, Mcp1, and Mip1α, but not Il-18) and an early hyperactivation of mTOR pathway (32). In addition, it has been shown that Nlrc3 is able to counteract CRC development by reducing c-Myc and the mTOR downstream targets FoxO3a and FoxO1 and inducing apoptosis (33). Moreover, *Nlrp12* deficiency significantly increased the colonic inflammation and tumorigenesis susceptibility through an increased production of pro-inflammatory cytokines and colonic activation of NF-kB, Erk, and Stat3 (34). In particular, generating bone marrow chimeras, the authors showed that *Nlrp12* in immune, rather than in epithelial cells, is critical for the protection against colitis and CAC and this effect appeared to be related to the Nlrp12-mediated suppression of canonical NF-kB and Erk in macrophages (34). Although a major role of canonical NF-kB signaling for CAC induction in *Nlrp12* deficient mice was proposed by Allen et al., a subsequent study emphasized the enhancement of non-canonical NF-kB signaling and MAPK activation as drivers of colonic inflammation and tumor-prone microenvironment derived from both hematopoietic and non-hematopoietic compartments (35). To explain these discrepancies, the authors speculated on the possibility that Nlrp12 may indirectly mediate canonical NF-kB signaling through the non-canonical pathway with a mechanism dependent on Ikkα and Traf3 (36). The importance of innate immunity in the regulation of inflammation and intestinal tumorigenesis was also extended to other non-forming inflammasomes NLR components, including Nod1. Chen et al. investigated the role of *Nod1* deficiency in models of colitis- and familial adenomatous polyposis-related carcinogenesis. The authors showed that *Nod1* deficiency alone or in combination with mutations in the Wnt signaling component *Apc*, in the context of inflammation, enhances tumor formation and progression by enhancing an inflammatory response that drives cell proliferation and malignant transformation (37). These results support the importance of NLR proteins in the prevention of intestinal inflammation and carcinogenesis.

## Role of NLR Proteins in Human IBD

Although functional studies aiming at clarifying the role of NLR proteins in the development of colitis and CAC have been mainly performed on pre-clinical models, observational and association studies conducted in humans partially support a protective role of some NLR proteins on IBD onset and progression. In particular, polymorphisms in genes encoding for NLR family members would confer a genetic predisposition or protection to IBD. Among NLR proteins, NOD2 (CARD15) has been investigated for a long time as susceptibility locus for Crohn’s disease (CD). Indeed, many studies led to establish that NOD2 loss-of-function variants are associated with an increased CD risk [for review see Ref. (38)]. In addition, the NOD2 missense variant R702W would enhance the CRC susceptibility in this population (39). Furthermore, NLRP3 has emerged as a predisposing gene for CD development, although, not all the studies give rise to robust results in different populations (40). Recently, further studies in humans have found low NLRP12 levels in ulcerative colitis (UC) patients (41), while NLRP6 upregulation has been observed in biopsy specimens from ileal CD patients (42). Importantly, despite studies on animal models suggested that reduced IL-18 levels, as downstream effect of NLR proteins alterations, might be critically responsible of increased susceptibility to colitis and CAC, data on humans showed opposite results. Indeed, increased IL-18 and IL-1β levels have been found in colonic tissues and cells from hematopoietic lineage from IBD patients [for review see Ref. (43)]. These observations led to hypothesize a causative role of the inflammasome hyperactivation in IBD pathogenesis and to question the translatability of the results obtained in inflammatory murine models in humans. Regarding the dual role of NLR proteins during inflammation, it has been suggested that increased IL-18 and IL-1β levels in the intestinal epithelial cells may be relevant to ensure a proper intestinal tissue regeneration and repair, while their induction in the lamina propria might trigger pro-inflammatory and deleterious responses (44).

Starting from the assumption that an aberrant activation of inflammasome may have a pivotal role in the development of IBD, therapeutic strategies aimed at repressing the activation of NLR proteins and thus reducing the levels of its functional mediators, have been proposed (45, 46). However, to date, data available on humans are limited to provide conclusive results.

Thus, to clarify the effective role of NLR system in the onset and the evolution of IBD, and to identify patients who can obtain a real benefit from therapies based on NLR proteins regulation, more studies will be necessary. For this purpose, it would be clinically relevant to stratify patients with IBD distinguishing between the type (CD vs. UC) and the duration (early- vs. long-term disease), to correlate colonic levels of inflammatory mediators with other markers of intestinal inflammation during IBD (i.e., fecal calprotectin) and to conduct more functional studies on intestinal organoids derived from IBD patients.

## NLR Proteins, Microbiota, and Risk for Colitis and Inflammatory-Driven CRC

The interest on the role of NLR proteins in the regulation of intestinal microbiota and its impact on the onset of intestinal inflammation and CAC have increased significantly over the past few years. Elinav and colleagues have first described this intriguing interplay. They demonstrated that, upon DSS treatment, mice with *Nlrp6, Asc*, or *Casp1* defect restricted to colonic epithelial cells were characterized by higher inflammation, intestinal hyperplasia, and reduced Il-18 levels. Intriguingly, this increased colitis susceptibility resulted to be transferable to co-housed *wt* mice, through a colitogenic microbiota characterized by a high prevalence of Prevotellaceae and TM7 bacterial taxa, particularly present in *Nlrp6* deficient mice (22). Moreover, *wt* mice co-housed with *Il-18* but not with *Il-1*β or *I1-1R* deficient mice had an increased susceptibility to DSS-induced colitis suggesting that the colonic defect in Il-18 is the major downstream event responsible of enhanced colitogenic microbiota in *Nrp6*^−/−^ mice. Moreover, a recent published work demonstrated that Nlrp6 was able to prevent colitis development in *Il-10*^−/−^ mice by modulating the abundance of *Akkermansia muciniphila* which may act as a pro-inflammatory bacterium (47). However, the role of *Akkermansia* in the pathogenesis of UC and CAC remains controversial. Indeed, we previously found an increase in the relative abundance of *Akkermansia muciniphila* in mice upon AOM-DSS treatment (48), while its depletion has been observed in long-term UC patients compared to healthy controls (49). Importantly, further studies, based on germ-free, antibiotics treated mice, as well as knockout animal models, have highlighted the importance of both commensal bacteria, microbial metabolites, and functional *Nlrp6* for the activation of the inflammasome, the induction of *Il-18* and the consequent production of anti-microbial peptides which is necessary to maintain a normal microbiota. Noteworthy, the administration of taurine to DSS-exposed mice, restoring an eubiotic microbiota, was associated with mucosal healing and improved survival (50). Accordingly, *Asc*^−/−^, *Nlrp6*^−/−^, and *Il-18*^−/−^, but not *Nlrc4*^−/−^ and *Il-1R*^−/−^ mice were found to be more prone to AOM-DSS-driven CAC (51).

Further studies have demonstrated that *Nlrp1b* deficiency in mice was associated with reduced colonic levels of Il-18 and Il-1β with a concomitant increased colitis susceptibility and CAC development upon DSS- and AOM-DSS treatment. Importantly, treatment with a broad-spectrum antibiotic mixture led to an amelioration of the phenotype (52).

Similarly, *Aim2*^−/−^ mice showed an impaired Il-1β and Il-18 synthesis associated with an overgrowth of colonic *E. coli* and an increased risk of developing colitis which may be counteracted by Il-18 infusion (53). In addition, a recent work demonstrated that deficiency for *Nlrp12* led to an increased colonic inflammation and caused a microbiota imbalance in mice by favoring the overgrowth of inflammatory bacteria, including members of *Erysipelothricaceae* family and inducing a concomitant reduction of beneficial strains such as members of *Lachnospiraceae* (41).

The protective effects of NLR signaling are also mediated by members of the NLR family that exert their function in immunity beyond the inflammmasome. Indeed, a Nod2-dependent protective effect of the strain *Lactobacillus salivarius* Ls33 has been described in a mouse model of 2,4,6-trinitrobenzene induced colitis (54). Simultaneously, a transmissible risk of colitis and CAC in *Nod2*-deficient mice transiently co-housed with *wt* mice in the context of AOM-DSS treatment has been observed in another study. Noteworthy, the *Nod2* mediated dysbiosis, following chemically induced injuries, impaired colonic epithelium integrity and generated an intestinal pro-inflammatory milieu with a critical role in the enhanced epithelial dysplasia. Importantly, this transmissible colitis risk was enhanced in germ-free *wt* hosts recolonized with dysbiotic fecal microbiota from *Nod2*-deficient mice, and conversely colonizing germ-free *Nod2*-deficient mice with *wt* microbiota reduced colitis severity, suggesting a reversible primary role of dysbiotic microbiota in intestinal tumorigenesis driven by inflammation (55). To support the idea that *Nod2* alterations could be directly linked with an abnormal composition of the microbiota, Ramanan et al. recently demonstrated that *Nod2* deficiency was responsible for the commensal *Bacteroides vulgatus* expansion in the small intestine which is transmissible to the next generation and responsible for goblet cells dysfunction, overproduction of Ifn-γ by intraepithelial lymphocytes and increased inflammation (56). A substantially altered microbial community structure in the terminal ileum of *Nod2*-deficient mice was also observed in another work, which contextually demonstrated that *Nod2* genotypes were associated with the microbial composition in humans (57). This effect in humans has also been pointed out by Knights et al., showing particularly an association between NOD2 risk allele count and increased relative abundance of Enterobacteriaceae (58). Conversely, a recent work demonstrated that the increased susceptibility to CAC observed in *Nod2*-deficient mice, might be due to a dysregulation of MAPK and NF-kB activation during inflammation, rather than the intestinal dysbiosis (59). Deficiency for *Card9* gene, an adaptor protein, acting downstream of *Nod2* on the MAPK pathway, confers an increased susceptibility to colitis and shows an altered gut microbiota profile and metabolism (60, 61).

Many studies that proposed a causal role of the inflammasome components in the induction of intestinal dysbiosis were conducted using non-littermate controls. Recent studies performed using littermate controls demonstrated that Nlrp6 deficiency does not directly affect the gut microbiota composition (62). Accordingly, previous studies aimed at evaluating the effect of Nod1 and Nod2 on microbiota using littermate controls revealed no direct effect of the genetic background on the gut microbiota composition (63, 64).

Although this is a complex issue in which both littermate status and mice separation need to be taken into account, these emerging findings suggest that non-genetic confounding factors such as maternal inheritance and the housing conditions may have critically influenced previous results obtained using non-littermate controls. Moreover, despite the fact that the pathogenic role of a dysbiotic microbiota in IBD has been widely demonstrated, only few studies investigated the hypothesis that changes in the relative abundance of specific bacterial populations could be driven by alterations of NLR proteins in humans. Indeed, to date, a causal role of NOD2 in the determination of ileal microbiota composition in CD patients has been proved [for review see Ref. (65)], while no consistent data regarding the impact of other NLR family components on microbiota structure, are currently available.

## Conclusion

**Figure 1:**
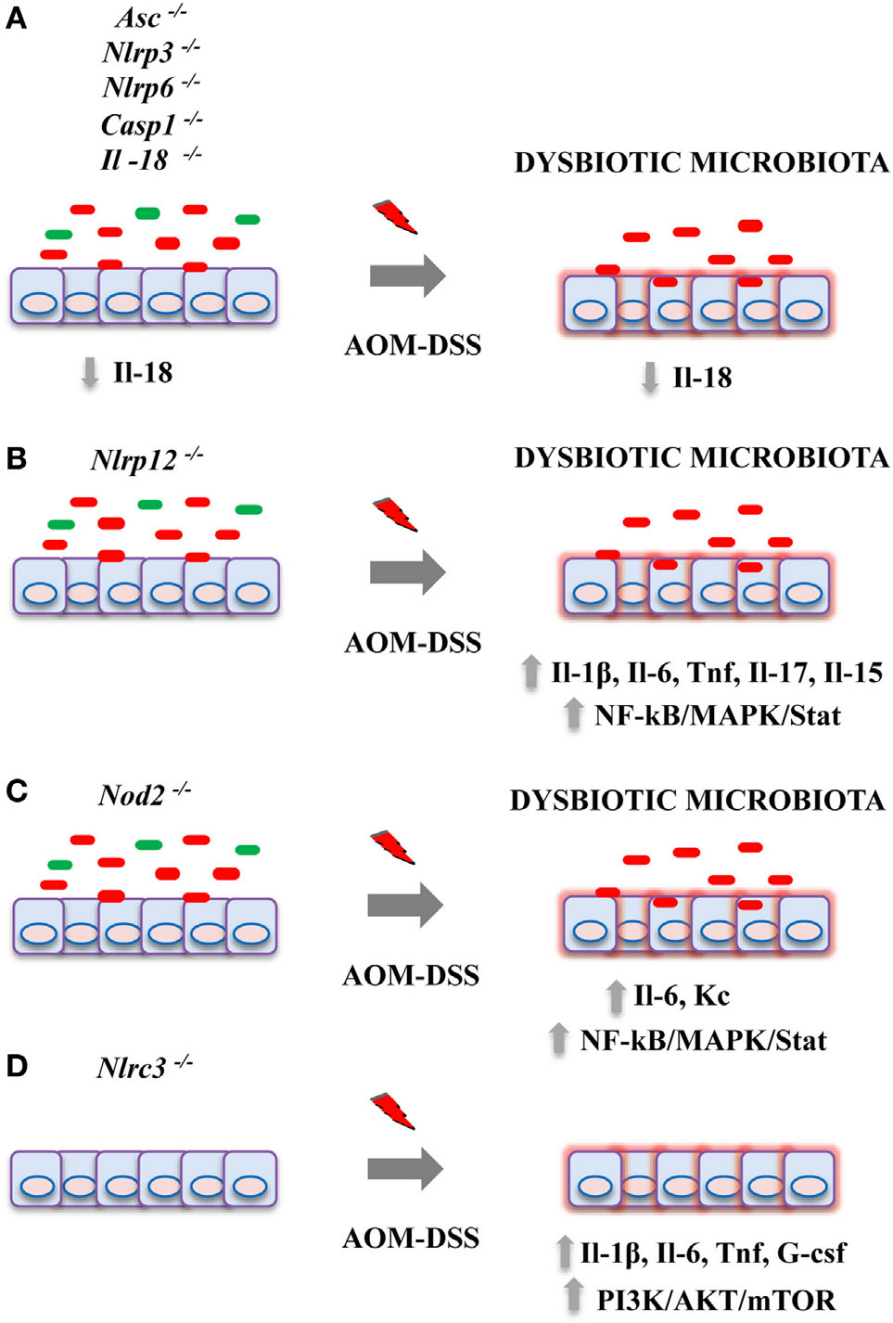
Effects of nucleotide-binding domain leucine-rich repeat containing (NLR) proteins deficiency on colitis-associated cancer (CAC) development in pre-clinical models. Genetic alterations in NLR proteins result in an increased tumor burden in azoxymethane (AOM)-dextran sulfate sodium (DSS) mouse model of CAC by acting on multiple mechanisms. **(A)** Deficiency for Asc, Nlrp3, Nlrp6, Casp1, or Il-18 genes led to a dysbiotic microbiota, reduced colonic Il-18 levels, and an impairment of intestinal tissue repair upon injury; **(B)** Nlrp12; and **(C)** Nod2 deficiency have been associated with an overgrowth of inflammatory bacteria, enhanced cytokine production, and activation of pro-tumorigenic pathways including NF-kB, MAPK, and Stat3. **(D)** Knockout for Nlrc3 led to increased colonic inflammation and mTOR signaling activation.

To date, there is growing evidence indicating that the NLR family components work as tumor suppressors in inflammation-induced tumorigenesis. Contextually, the crucial role of the intestinal microbiota during the onset and development of CAC has been proved (66–68). The important emerging concept is that alterations of NLR protein functions and a dysbiotic microbiota are not distinct phenomena during the genesis of colitis and CAC, but rather closely connected events in a causal mutual relationship (see Figure 1 for a schematic model on effects of NLR proteins deficiency on CAC development). Improper activity in the intestinal immune system due to the loss of function of specific NLR receptors during the exposure to inflammatory stimuli might support (or lead to) the overgrowth of inflammatory bacteria enhancing intestinal tumorigenesis. This observation indicates that by modulating the composition of the gut microbiota we could possibly counteract the deleterious effects of an unfavorable genetic predisposition. However, it is important to underline that this experimental evidence is mainly derived from animal models and the translation of these observations in patients with IBD or CAC will be necessary. In addition, some of these results were not confirmed in studies conducted using littermate controls. In light of this new evidence, it will be necessary to reconsider previous obtained findings reducing the impact of confounding factors. Moreover, a number of relevant questions is still open: (1) What is the impact of NLR proteins on human intestinal microbiota in patients with IBD? (2) Can we really define a “colitogenic” microbiota during defective NLR signaling? (3) Could we use microbial changes to early detect alterations in NLR protein families? and (4) Can we prevent CAC development improving the protective NLR functions during long-standing IBD?

In conclusion, although the role of the microbiota in supporting a tumor-prone microenvironment in a context where the protective immunity is defective seems crucial, it is necessary to best characterize the microbial species able to exacerbate intestinal inflammation and cancer when specific NLR family components were defective. Moreover, the validation of the functional effects of microbiota changes discovered through high-throughput analysis may clarify its role in the diseases allowing the development of promising approaches for the prevention and treatment of colitis and CAC.

## Author Contributions

AP and LR retrieved and analyzed concerned literatures and wrote the manuscript. HS revised the manuscript for important intellectual content. All authors contributed to manuscript revision, read and approved the submitted version.

## Conflict of Interest Statement

LR has received an unrestricted research grant by SLA Pharma and Takeda. No potential conflict of interest was disclosed by the other authors.

## Abbreviations

AOM, azoxymethane; CAC, colitis-associated cancer; Casp1, Caspase1; CLRs, C-type lectins receptors; CD, Crohn’s disease; DAMPs, damage-associated molecular patterns; DSS, dextran sulfate sodium; IBD, inflammatory bowel disease; NLR, nucleotide-binding domain leucine-rich repeat containing; PAMPs, pathogen-associated molecular patterns; PRRs, pattern recognition receptors; RLRs, retinoic acid-inducible gene (RIG)-I-like receptors; TLRs, toll-like receptors; UC, ulcerative colitis; wt, wild-type.
